# Bioenergetics modeling of the annual consumption of zooplankton by pelagic fish feeding in the Northeast Atlantic

**DOI:** 10.1371/journal.pone.0190345

**Published:** 2018-01-02

**Authors:** Eneko Bachiller, Kjell Rong Utne, Teunis Jansen, Geir Huse

**Affiliations:** 1 Pelagic Fish Research Group, Institute of Marine Research (IMR), Bergen, Norway; 2 GINR–Greenland Institute of Natural Resources, Nuuk, Greenland; 3 DTU Aqua–National Institute of Aquatic Resources, Technical University of Denmark, Charlottenlund Castle, Charlottenlund, Denmark; Technical University of Denmark, DENMARK

## Abstract

The present study uses bioenergetics modeling to estimate the annual consumption of the main zooplankton groups by some of the most commercially important planktivorous fish stocks in the Northeast Atlantic, namely Norwegian spring-spawning (NSS) herring (*Clupea harengus*), blue whiting (*Micromesistius poutassou*) and NEA mackerel (*Scomber scombrus*). The data was obtained from scientific surveys in the main feeding area (Norwegian Sea) in the period 2005–2010. By incorporating novel information about ambient temperature, seasonal growth and changes in the diet from stomach content analyses, annual consumption of the different zooplankton groups by pelagic fish is estimated. The present study estimates higher consumption estimates than previous studies for the three species and suggests that fish might have a greater impact on the zooplankton community as foragers. This way, NEA mackerel, showing the highest daily consumption rates, and NSS herring, annually consume around 10 times their total biomass, whereas blue whiting consume about 6 times their biomass in zooplankton. The three species were estimated to consume an average of 135 million (M) tonnes of zooplankton each year, consisting of 53–85 M tonnes of copepods, 20–32 M tonnes of krill, 8–42 M tonnes of appendicularians and 0.2–1.2 M tonnes of fish, depending on the year. For NSS herring and NEA mackerel the main prey groups are calanoids and appendicularians, showing a peak in consumption during June and June–July, respectively, and suggesting high potential for inter-specific feeding competition between these species. In contrast, blue whiting maintain a low consumption rate from April to September, consuming mainly larger euphausiids. Our results suggest that the three species can coexist regardless of their high abundance, zooplankton consumption rates and overlapping diet. Accordingly, the species might have niche segregation, as they are species specific, showing annual and inter-annual variability in total consumption of the different prey species. These estimates and their inter-annual and inter-specific variation are fundamental for understanding fundamental pelagic predator-prey interactions as well as to inform advanced multispecies ecosystem models.

## Introduction

The Northeast Atlantic has extensive oceanic areas with high zooplankton densities in the upper waters during summer. The area is therefore a major feeding area for some of the largest planktivorous fish stocks in the world, including Norwegian Spring Spawning (NSS) herring (*Clupea harengus*, Linnaeus, 1758), blue whiting (*Micromesistius poutassou*, Risso 1827) and Northeast Atlantic (NEA) mackerel (*Scomber scombrus*, Linnaeus, 1758). Among these pelagic planktivorous stocks, while NSS herring spend their life cycle within the Norwegian Sea and Barents Sea [1,2], NEA mackerel and blue whiting overwinter and spawn elsewhere in the Northeast Atlantic, but migrate into the Norwegian Sea to feed during spring and summer [3]. Consequently, these three pelagic stocks show a substantial spatial [4,5] and dietary [6–8] overlap in the Norwegian Sea, their main feeding ground during these seasons. Their diet consists mainly of various zooplankton species with the copepod *Calanus finmarchicus* as the most important prey [6–8]. All three stocks have had large fluctuations in stock size the last decades, mainly due to variable recruitment and fishing pressure [9]. There has been a substantial increase in the total biomass of these stocks since the 1980s, and during the last decade it has been about 15 million (M) tonnes [9].

The total annual production of *C*. *finmarchicus* in the Norwegian Sea is estimated to be in the range of 200–300 million tonnes [1,10], but the uncertainty is high. In addition to small pelagic fish, there is a range of other predators including other zooplankton species that prey on zooplankton, small mesopelagic fish and whales. Large zooplankton like krill and amphipods as well as squids are estimated to consume around 150–200 M tonnes of *C*. *finmarchicus* [1]. The annual consumption of zooplankton by pelagic fish in the Norwegian Sea is an issue that has been the focus of several studies [1,11–13]. In fact, planktivorous fish populations can be very abundant and have a great impact on the ecosystem [14,15], e.g. reducing the zooplankton biomass in restricted marine areas such as the southeast Bering Sea [16], the Baltic Sea [17], the Black Sea [18] and the Barents Sea [14,19–21].

Knowledge of the zooplankton consumption by planktivorous fish stocks is therefore important for several reasons. Regarding the energy flow from one trophic level to another [22], the zooplankton community is key to understand the bottom-up control in the Nordic Seas (e.g. [23,24]), usually underestimated (e.g. [25]), and has received an increased scientific interest during the last decade [3,26]. In addition, there are two important management issues that are in need of knowledge addressing trophic regulation in the Norwegian Sea. The first question is whether there is enough zooplankton available for the large fish stocks feeding in the area [3], considering the increase in fish biomass and changes in the zooplankton community during the last decades [1,9,27]. There has been reduced individual growth and increasing stock size for all three fish stocks [3]. There is density dependent regulation of both juvenile [28] and adult [28,29] mackerel, where both interspecific competition with herring as well as intraspecific competition affect the individual’s growth [3,28,29]. The competition for food has likely been one of the key drivers for the expansion of the feeding area towards north and west [30].

The second question is related to a new fishery based on pelagic trawling directly targeting spawning components of the copepod *C*. *finmarchicus* in eastern part of the Norwegian Sea. This fishery is presently small scale, but is expected to increase in quantity and geographic extent the coming years [31]. The total allowable catch for harvesting *C*. *finmarchicus* has recently been increased from 1000 t to 165 000 t as part of the development of a management plan [32,33]. An important concern is whether this fishery will increase the negative effect on individual growth for pelagic fish, as it becomes a direct competitor for *C*. *finmarchicus*. It will therefore be important to expand the knowledge base on zooplankton consumption exerted by the planktivorous stocks prior to a potential increase in *Calanus* harvest.

To estimate the annual consumption of zooplankton several approaches can be used, ranging from simple assumptions about consumption/biomass ratios, as used by Dommasnes et al. [12], to more complex approaches including coupled individual based models [13]. However, previous estimates are limited to total zooplankton consumption, whereas detailed consumption of different prey groups is still unknown. This is important when observing potential competition for food between co-occurring fish populations such as NEA mackerel, NSS herring and blue whiting, which show interspecific differences in the relative importance of different prey ingestion [8]. In this sense, bioenergetics modeling is an efficient method for estimating annual consumption, as field estimates of food consumption are often highly variable and require considerable effort [34]. Energy budgets and energetic models, in combination with field data on fish growth and water temperature, are important tools for predicting food consumption [24,35,36]. The approach is to estimate the total consumption as equal to the sum of all energy expenses for the individual fish. This consists mainly of fish growth, change in energy content (mainly fat) through the year [37], and metabolic costs. When combined with observations of dietary composition of the fish and energy density of the food organisms, the annual consumption of different prey groups can be estimated.

The objective of this study is to estimate the annual food consumption for NEA mackerel, NSS herring and blue whiting using bioenergetics modeling with species-specific parameters for the years 2005–2010. The present study makes a novel approach to such estimates, considering 1) recent diet composition information for the three species [8], 2) the length-growth during the feeding season in the Norwegian Sea–which was omitted by previous studies (e.g. [11,13])–, 3) new energy density estimates for NEA mackerel, and 4) metabolic costs accounting for new ambient temperature measurements representing the horizontal and vertical distribution of the species. The results are discussed both in an ecological context and compared to consumption estimates from previous studies. The consumption estimates are for the entire stocks independent of where feeding takes place, although most of the fish are feeding in the Norwegian Sea [8]. A sensitivity analysis is also provided as the consumption estimates strongly depend on the input data and parameters applied in the model [38].

## Material and methods

### Bioenergetics model purpose: Consumption estimates

Fish require a certain amount of energy for swimming, feeding, growing and reproducing throughout the year. Bioenergetics models calculate the energy needed for respiration (including activity costs and specific dynamic action), waste losses (egestion and excretion) and growth [39]. In this study a bioenergetics model is applied for the NEA mackerel, NSS herring and blue whiting in order to estimate their annual consumption of different prey groups defined from their diet composition [8] for the years 2005–2010. With this model, both monthly and inter-annual variations can be calculated for the three species. The annual consumption is split into seven prey groups based on stomach content information and the energy demand throughout the feeding period. The simulations apply biomass estimates from the analytic assessment in 2015 [9] to estimate the total consumption of the stocks.

### Sampling and data range definition

**Fig 1:**
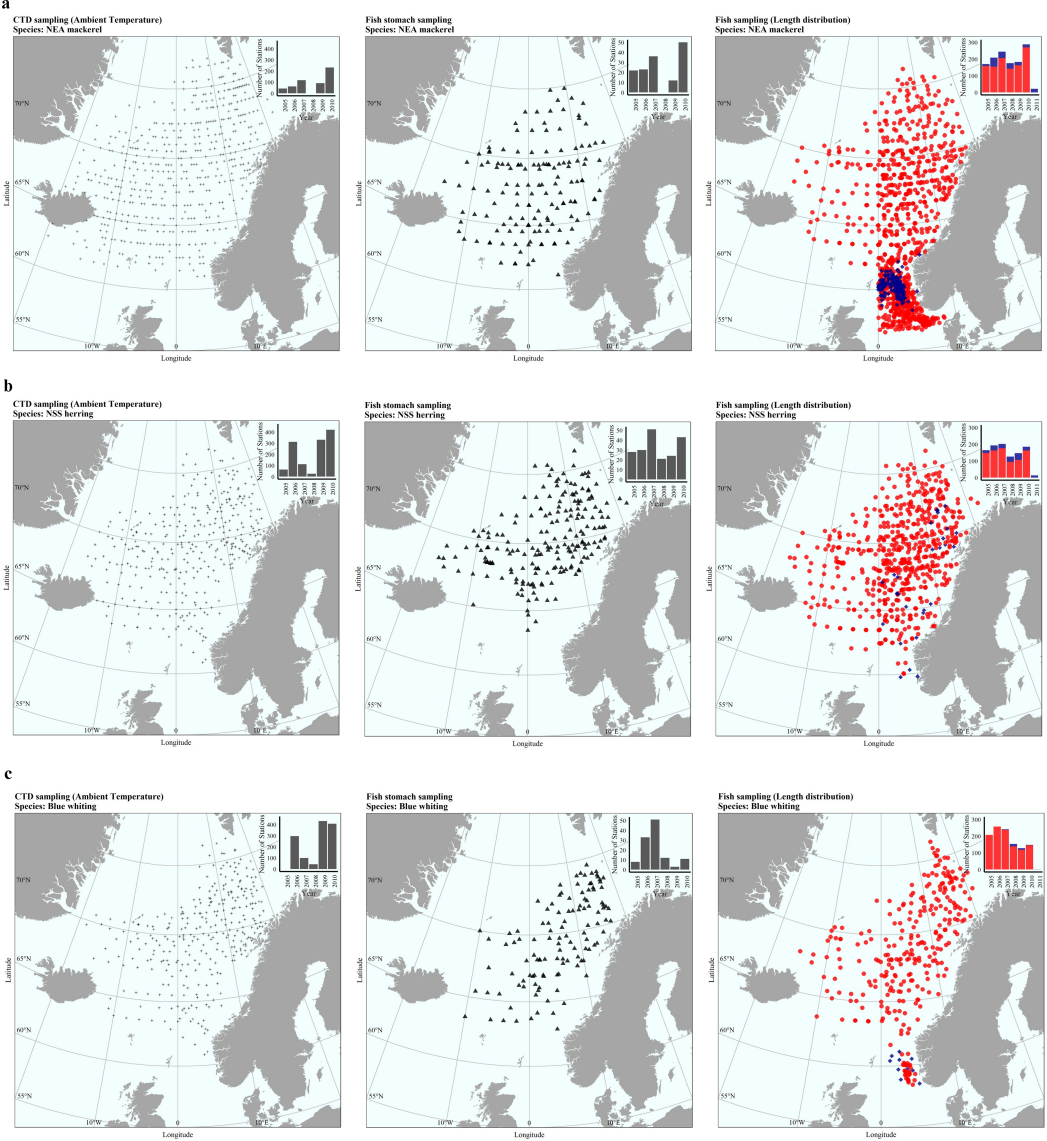
**Map of samples of (a) NEA mackerel, (b) NSS herring and (c) blue whiting, used to get different information used as input for the analysis.** Small dots represent CTD sampling stations considered for ambient temperature calculations. Triangles represent stations used for the diet characterization analysis [8]. Fish length and weight measurements used as input for the growth in the bioenergetics consumption estimation model are from summer stations and represented with red circles, whereas fish collected during winter time (i.e. no growth) from commercial vessels are from stations marked with blue rhombus. Note that many stations (considered in bar charts in the upper right corner of each plot and in Table 1) are not indicated in maps. These are mostly winter sampling stations from commercial vessels not providing detailed position information. During winter time herring were distributed in the Norwegian Sea, whereas mackerel and blue whiting were generally collected southwards, in the southern region of the Norwegian Sea as well as in the North Sea.

All the analyses are based on data from Norwegian pelagic ecosystem surveys as well as from commercial vessels fishing in the area, from 2005 to 2010 (Fig 1; Table 1). The monitoring surveys are the International Ecosystem Survey in the Nordic Seas (IESNS) in May and the International Ecosystem Summer Survey in the Nordic Seas (IESSNS) in July/August. These surveys provided Conductivity, Temperature and Depth (CTD) measurements, acoustic data, trawl samples, morphometric measurements (length and weight) and stomach content of fish. CTD casts were carried out using Seabird 911 and SAIV SC 204 instruments from the surface down to 500 m, and were taken prior to the fish trawls. The acoustic data, given as Nautical Area Scattering Coefficient (NASC, [40]), were collected using Simrad EK60 with a calibrated 38 kHz split beam echo sounder and stored with a resolution of 10 m vertically and 1 nm horizontally. The raw data were scrutinized during the surveys and the acoustic values were assigned to species based on appearance on echograms and the observed composition in trawl hauls. Stomach content information comes from the study by Bachiller et al. [8], that investigated the diet based on sampling in May and July 2005–2010 (Fig 1; Table 1). Since summer is the main feeding period of NEA mackerel in the Norwegian Sea [3,8,41], no mackerel was caught in May surveys and therefore diet composition information for this species was only available for July (see Bachiller et al. [8] for further details).

**Table 1:** Sampling stations (N_st_) and number of fish (N_f_) per species considered for each purpose during the study. ‘ac’ means acoustic measurements (i.e. no fish sampled).

			NEA mackerel (Age groups: 2–17)		NSS herring (Age groups: 4–17)		Blue whiting (Age groups: 2–13)	
Sampling purpose	Year	Sampling period	N_st_	N_f_	N_st_	N_f_	N_st_	N_f_
Ambient temperature: fish + CTD sampling	2005	May 01 –July 29	44	ac	63	ac	-	ac
	2006	May 01 –Aug 03	62	ac	313	ac	296	ac
	2007	May 01 –Aug 03	118	ac	112	ac	99	ac
	2008	May 06 –Aug 07	-	ac	26	ac	43	ac
	2009	May 01 –Aug 04	91	ac	331	ac	431	ac
	2010	May 07 –Aug 18	266	ac	421	ac	407	ac
Length distribution (winter): fish sampling	2005	Sep 17 –Oct 29	12	516	17	811	-	-
	2006	Sep 21 –Oct 24	53	1339	30	831	-	-
	2007	Sep 18 –Oct 27	38	916	26	672	3	11
	2008	Sep 16 –Oct 24	33	955	32	928	14	155
	2009	Sep 18 –Oct 15	19	574	40	1286	10	65
	2010	Sep 19 –Oct 30	18	499	22	636	3	95
	2011	Sep 16 –Oct 20	22	656	14	410	1	2
Length distribution (growth period): fish sampling	2005	May 12 –Sept 03	158	6986	148	5630	207	14538
	2006	Apr 03 –Sept 20	155	5479	163	8110	257	15973
	2007	Apr 27 –Sept 16	206	7437	176	9357	238	15472
	2008	May 05 –Sept 14	142	4025	94	4207	139	9346
	2009	Apr 04 –Sept 16	163	5912	107	3105	118	7339
	2010	Apr 08 –Sept 14	270	13461	163	9105	145	6174
Diet composition: fish sampling	2005	May 01 –July 29	22	212	28	265	8	80
	2006	May 01 –Aug 03	23	229	30	299	33	313
	2007	May 01 –Aug 03	36	346	51	500	51	495
	2008	May 06 –Aug 07	-	-	21	163	12	111
	2009	May 01 –Aug 04	12	71	24	162	3	30
	2010	May 07 –Aug 18	50	499	43	410	11	107

All the data underlying the present study are available from the Dryad Digital Repository (doi: 10.5061/dryad.gb786).

As all three stocks spawn and have nursery areas outside the Norwegian Sea, NEA mackerel usually enter the Norwegian Sea as 2 year old [42], herring as 4 year old [1] and blue whiting as one year old [9]. Accordingly, the length ranges considered in the study were those corresponding to the size fractions that are feeding in the Norwegian Sea: 25–45 cm for NEA mackerel, 28–38 cm for NSS herring and 15–40 cm for blue whiting. Since Bachiller et al. [8] studied the prey composition of 32–41 cm mackerel, 29–34 cm herring and 28–32 cm blue whiting, their diet composition information fits well with these pre-defined length ranges.

Information about change in length and weight throughout the feeding season is needed to account for growth and stored energy in the model. Accordingly, length–weight measurements within the pre-defined length ranges provided by commercial fishing vessels (Fig 1; Table 1) were also included in the analyses.

**Fig 2:**
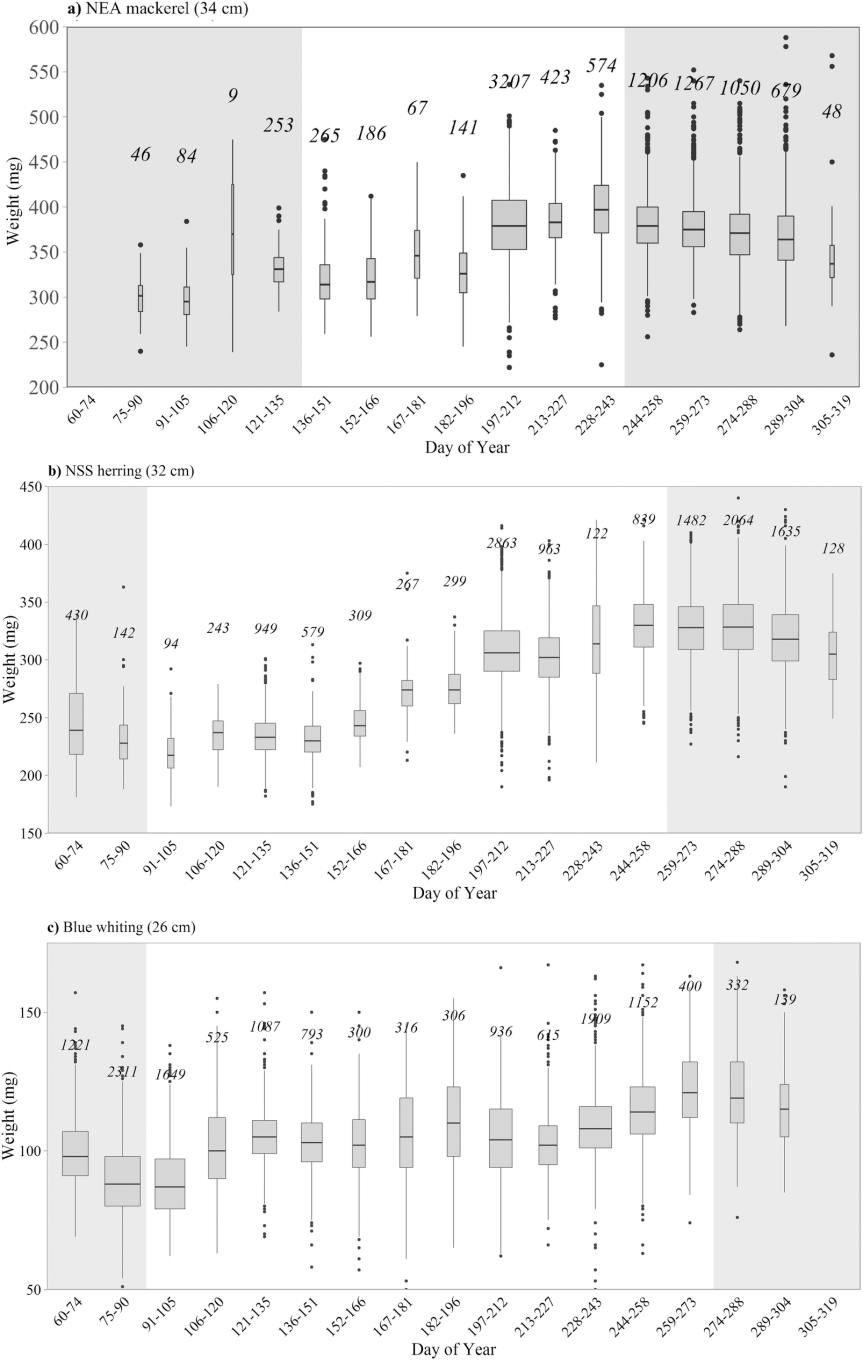
**Box-plots of the average weight (mg) in 15-day periods for (a) 34 cm NEA mackerel, (b) 32 cm NSS herring and (c) 26 cm blue whiting, considering data from all the years (2005–2010) together.** The width of the boxes is proportional to the sample size (number of measurements are indicated above each box-plot). White background represents the growing period considered for the analyses.

NEA mackerel, NSS herring and blue whiting grow during the feeding season [13,29,43], whereas during winter the feeding nearly stops and the length growth drops down significantly [1,44]. The feeding period was defined as the period when the fish increased the weight at length. This was calculated from the change in weight per length, using the most frequent length group in the samples from the surveys. These were 34, 32 and 26 cm length groups for NEA mackerel, NSS herring and blue whiting, respectively. This analysis is presented in Fig 2 and shows how the feeding period was defined: from May 16^th^ (Day of Year = 136) to August 31^st^ (Day of Year = 243) for NEA mackerel; from April 1^st^ (Day of Year = 91) to September 15^th^ (Day of Year = 258) for NSS herring; and from April 1^st^ (Day of Year = 91) to September 30^th^ (Day of Year = 273) for blue whiting.

### Model design concepts

The purpose of the bioenergetics model is to estimate the annual consumption of different prey groups during the feeding season. The model calculates the consumption for each fish species and length group and scales it to population level, based on the length distribution in the respective populations. In this sense, consumption was modelled according to a standard bioenergetics model with species specific parameter values [37], based on the following equation:

C=R+F+E+S+G (1)

*C* is consumed food in g prey g predator^-1^ day^-1^. *R* is energy loss due to respiration (g prey g predator^-1^ day^-1^) and depends on body mass (g wet weight), ambient temperature and swimming speed. *F* and *E* are energy loss due to egestion and excretion, respectively, and *S* is energy loss due to specific dynamic action. *G* is growth (g prey g predator^-1^ day^-1^) and is a function of changes in fish weight and energy obtained during the feeding season. Consumption, respiration, specific dynamic action, excretion, and egestion are converted to g fish g fish^−1^ d^−1^ by the ratio of the prey energy densities.

The model is run for the feeding period in the spring and summer with daily time steps, where the number of days of the period is species specific. Input data to the model, such as ambient temperature and prey composition, is updated each time step based on survey observations. The basics of the model are the same as those used in previous similar studies [11,13]. Although it still assumes no mortality, the present model provides new insights in several aspects:

1. Updated diet composition information, now available for different seasons [8], allows estimating the energetic income from different species. The daily consumption is estimated for 7 different prey groups. This will lead to more precise estimates of annual zooplankton consumption by the NEA mackerel, NSS herring and blue whiting.
2. Since growth is an integrator of consumption over time, bioenergetics models can be used to derive consumption estimates based on observed growth over a defined period [37]. In contrast to previous studies for these species in the Norwegian Sea [11,13], this study considers the daily length–growth during the feeding migration.
3. In the short feeding season, there is also a change in the energy content of the fish. In this sense, seasonal cycles in the energy density of the predator can strongly influence estimated seasonal consumption patterns [11,45,46]. Changing energy density of the fish is a method to store energy independent of somatic growth. However, this has not been included in previous consumption estimates. Varpe et al [11] included changes in energy density when calculating the energy needed for weight gain, but not the energy needed to increase the energy density of the existing body mass. In this sense, our model considers daily changes in energy density of the fish.
4. Water temperature affects fish metabolism and consumption rates [3,13,47,48]. This study combines survey data of spatial (i.e. horizontal and vertical) distribution of fish with water temperature measurements from CTDs, to calculate ambient temperature for two periods during the feeding season. In this sense, ambient temperature estimates are more accurate than in previous studies [4,8,11,13,49–51].

### Model input

#### Swimming speed

The swimming speed of NEA mackerel, NSS herring and blue whiting was defined as in Varpe et al. (2005), being one body length per second.

#### Ambient temperature

For NSS herring and blue whiting, the ambient temperature (*aT*) each year (2005–2010) was calculated for May and July separately, according to the following equation:

$$aT=\frac{\sum_{st=1}^{n}\sum_{d=5}^{m}T_{st,d}{SA}_{st,d}}{\sum_{st=1}^{n}\sum_{d=5}^{m}{SA}_{st,d}}$$ (2)

Since acoustic data were averaged and projected into 1° latitude by 1° longitude grids, *st* corresponds to the average value of each grid or stratum. *T*_*st*,*d*_ is the water temperature at stratum *st* and depth *d*. Depth is defined in 10 m bins from the surface to the maximum depth *m* with available acoustic data. *SA*_*st*,*d*_ is the estimated fish abundance (see ‘Sampling and data range definition’ section) at stratum *st* and depth *d*. In those cases where more than one CTD measurement was obtained for the same stratum (*st*), the average temperature per depth (*d*) was considered.

During the feeding season NEA mackerel is concentrated in the upper water layer in loose shoals [52], making standard acoustic methods unreliable for abundance estimation [53]. Instead, catch–per–unit–effort (CPUE) in kg m^-2^ from standardized surface trawling was used as a proxy for the total NEA mackerel abundance at stratum *st*, and 10 m depth as a fixed depth (*d*) for NEA mackerel vertical distribution.

The ambient temperature estimated for May was used as input in the model before June 1^st^. After July 1^st^ the ambient temperature for July was used. For dates in between, a linear interpolation between May and July data was used.

#### Diet composition and energy density of prey groups

For the bioenergetics model, stomach contents described in Bachiller et al. [8] were categorized into 7 prey groups: Copepoda subcl. (all copepods grouped), Euphausiacea ord., Amphipoda ord., other crustaceans (crustaceans not included in previous groups), Appendicularia cl., Actinopterygii cl. and other remains (other prey).

Prey energy densities (J g^-1^ wet weight) were used to convert consumption from Joule to prey biomass. The energy density of copepods was set to 3600 J g^-1^ for *C*. *finmarchicus* [11,13,37,54]. For euphausiids and amphipods an energy density of 4000 J g^-1^ was used [37,55]. Fish were rarely found in stomachs and were not identified to species level [8]. However, a preliminary data review made for the present study determined that the identified fish organisms consisted mainly of herring larvae [56] and small mesopelagic fish (e.g. *Maurolicus muelleri* [57]). The energy density of adult NSS herring estimated in this study (see in Material and Methods section below) ranged between 8270 and 15440 J g^-1^, and values of mesopelagic fish could vary between 7490 and 10250 J g^-1^ from small to large specimens respectively [58]. Therefore, and considering the small size of fish observed in stomach contents, a conservative value of 8500 J g^-1^ was applied for this group. For the remaining prey groups the same energy density as for *C*. *finmarchicus* was used, as in previous studies [11,13].

Prey composition in percentages for each predator species, season and year used as input for the model is presented in Table 2. There was no available information of prey composition for NEA mackerel in May, or for NSS herring and blue whiting in July some years. The NEA mackerel prey composition for July was used for the entire feeding season. In years without July information for NSS herring and blue whiting, the average value obtained from the other years was applied (Table 2). As for ambient temperature, prey composition for May was used as input in the model before June 1^st^. After July 1^st^ the diet for July was used. To consider the daily change in the diet composition for dates in between, a linear interpolation in prey group proportions between May and July was used.

**Table 2:** Diet composition (prey percentages) for NEA mackerel (*S*. *scombrus*), NSS herring *(C*. *harengus*) and blue whiting (*M*. *poutassou*) in the Norwegian Sea (2005–2010). Modified from Bachiller et al. [8].

	Year	Season	Copepoda subcl.	Euphausiacea ord.	Amphipoda ord.	Other crustaceans	Appendicularia cl.	Actinopterygii cl.	Other remains
NEA mackerel	2005	July	72	4	4	1	14	1	4
	2006	July	89	2	<1	4	4	0	2
	2007	July	67	<1	2	0	30	0	1
	2008	July*	61	6	2	11	18	<1	3
	2009	July	26	12	1	50	9	0	2
	2010	July	50	11	1	<1	31	1	6
NSS herring	2005	May	91	3	1	0	5	0	<1
		July	73	10	2	5	9	0	<1
	2006	May	92	7	<1	0	1	0	<1
		July*	53	13	10	2	21	0	<1
	2007	May	41	7	4	1	47	0	1
		July*	53	13	10	2	21	0	<1
	2008	May	42	6	3	1	48	<1	<1
		July	31	6	3	<1	60	0	0
	2009	May	61	4	<1	0	32	0	3
		July	48	30	22	0	<1	0	<1
	2010	May	82	2	4	0	0	0	13
		July	61	8	12	4	15	0	1
Blue whiting	2005	May	73	23	3	0	<1	0	0
		July*	17	63	19	1	1	0	<1
	2006	May	33	48	6	0	7	2	4
		July*	17	63	19	1	1	0	<1
	2007	May	33	55	7	0	0	3	2
		July	2	88	8	0	1	0	1
	2008	May	39	46	3	0	0	11	<1
		July*	17	63	19	1	1	0	<1
	2009	May	0	93	6	0	0	0	0
		July*	17	63	19	1	1	0	<1
	2010	May	4	67	26	0	0	2	1
		July	32	38	29	1	0	0	0

(*) No available diet information, so the average between values for the same prey species obtained for the other applicable years was applied.

#### Abundance distribution per length (in winter)

Historic fish abundance was calculated from number-at-age and weight-at-age from the analytic assessment reported by ICES WGWIDE in 2015 [9]. Total number-at-age was applied without attempting to quantify the proportion of fish feeding in the Norwegian Sea. As the bioenergetics model is length specific, it was necessary to express the stock as number of individuals per 1 cm length group. Accordingly, the average length at age–specific weight (*L*) was calculated based on the following length–weight relationship:

$$W=aL^{b}$$ (3)

where *W* is the weight in grams [9] and *a* and *b* are constant parameters from regression equations (Table 3).

**Table 3:** Length (*L*)–Weight (*W*) relationships used to calculate the total length at age for NEA mackerel, NSS herring and blue whiting.

Species	Country	Area	N_f_	LR (cm)	a	b	R^2^
NEA mackerel	Ireland [59]	Celtic Sea	1801	15–44	0.00338	3.241	0.996
NSS herring	Various [60]	North Sea; ICES sub-area IV, Divisions VIId and IIIa	20165	7–37	0.00322	3.22	0.991
Blue whiting	France [59]	Bay of Biscay	1272	14–40	0.00375	3.082	0.992

N_f_ is the number of fish samples from a certain length range (*LR*), collected by different countries in different areas, used for the exponential regression equations, Eq (3).

The total number of individuals in each length group is calculated by assuming a Gaussian distribution using estimated variance from samples taken from winter commercial catches in the Norwegian Sea (Fig 1; Table 1). Hence, the standard deviation (SD) in each age group, year and species was firstly calculated from these data. Then, fish numbers at age obtained from ICES [9] for each year were re-distributed to 0.1 cm length groups, following a normal distribution with the calculated SD. This way, new abundance estimates for each cm length group were obtained excluding age information. As the last step, the length distribution and the total biomass estimated for the start of the feeding period were scaled to the annual biomass estimates from the assessment [9], according to the following equation:

$${ABD´}_{L}={ABD}_{L}\left(\frac{B_{ICES}}{\sum_{L_{min}}^{L_{max}}W_{L,t=91}{ABD}_{L}}\right)$$ (4)

where *ABD'*_*L*_ is the resulting abundance distribution used as input for the model. *ABD*_*L*_ denotes numbers of fish from *L* cm length group. *B*_*ICES*_ is the total annual biomass from the assessment [9]. For NEA mackerel and blue whiting total stock biomass estimates were used, whereas for NSS herring the spawning stock biomass was used. *W*_*L*,*t = 91*_ is the total weight of fish of *L* cm, at the beginning of the feeding period (*t* = 91; see ‘Sampling and data range definition’ section for definition of *t* and the next section for *W* calculation equation).

#### Somatic growth and change in energy content

Somatic growth during the feeding season is the combined effect of length growth and changes in weight-at-length. To calculate the weight-at-length, the length data from the feeding season (Table 1) were grouped into 1 cm groups and second order polynomial equations were fitted to the data, combining all sampling years (2005–2010):

$$W_{L,t}=a_{L}t^{2}+b_{L}t+c_{L}$$ (5)

where *W*_*L*,*t*_ is the weight (in grams) at a certain day (*t*, in Day of Year) and *a*, *b* and *c* are constant regression parameters specific for each length group (*L*).

Then, the length increment per year was estimated using the following growth model [61], which is a modification of Von Bertalanffy’s growth equation without the time dimension:

$$dL=k(L_{max}-L_{s})$$ (6)

This model assumes that the length increment of fish (*dL*) living under constant conditions and unlimited food supply decreases linearly with increasing total length of fish (*L*_*s*_), until it reaches zero at a maximal fish length (*L*_*max*_); *k* is a variable determined by environmental factors, such as food availability and temperature [61]. Based on winter (December-February) length-at-age measurements, a *k* value was estimated from the increment in length of each age-group from year *x* to year *x+1*. The average *k* (i.e., all age-groups together) per year and species was then calculated and used as input for the model (Table 4).

**Table 4:** Estimated average *k* values from Eq (6), considering the increment in length (*dL*, from winter in year *x* to winter in year *x+1*) and the maximum length for growth (*L*_*max*_) assumed for each species.

	NEA mackerel	NSS herring	Blue whiting
***L***_***max***_ **(cm)**	42	36	35
***k***			
**2005**	0.34	0.15	0.19*
**2006**	0.24	0.15	0.19*
**2007**	0.47	0.19	0.19*
**2008**	0.36	0.17	0.26
**2009**	0.19	0.17	0.07
**2010**	0.16	0.24	0.24

(*) Since there was no available information for these cases, the average between values for the other years was applied.

The growth of the modeled fish is in discrete centimeters to limit the time required for each simulation. As individual growth is a continuous process, and assuming a linear growth in length through the feeding season, a growth adjustment had to be done in the model. The weight at the start of the feeding season was calculated from Eq (5). The estimated *dL* for each length-group in Eq (6) was split in upper (*dG*_*upper*_) and lower (*dG*_*lower*_) integer values, corresponding to the values obtained when rounding *dL* to the nearest upper and lower absolute (cm) values, respectively. The relative difference between the lower bound (*dG*_*lower*_) and *dL* was defined as *RdL*_*lower*_, whereas the relative increment from *dL* to the upper bound (*dG*_*upper*_) was defined as *RdL*_*upper*_. The total weight of the individual at the end of the feeding season was given by:

$${W^{\prime }}_{t,L_{s}}=\left(W_{t,L_{s},dG_{upper}}RdL_{upper}\right)+\left(W_{t,L_{s},dG_{lower}}RdL_{lower}\right)$$ (7)

where *W’*_*t*,*Ls*_ is the fish weight of length group *L*_*s*_ at the end of the feeding season (*t* in Day of Year, see ‘Sampling and data range definition’ section).

**Fig 3:**
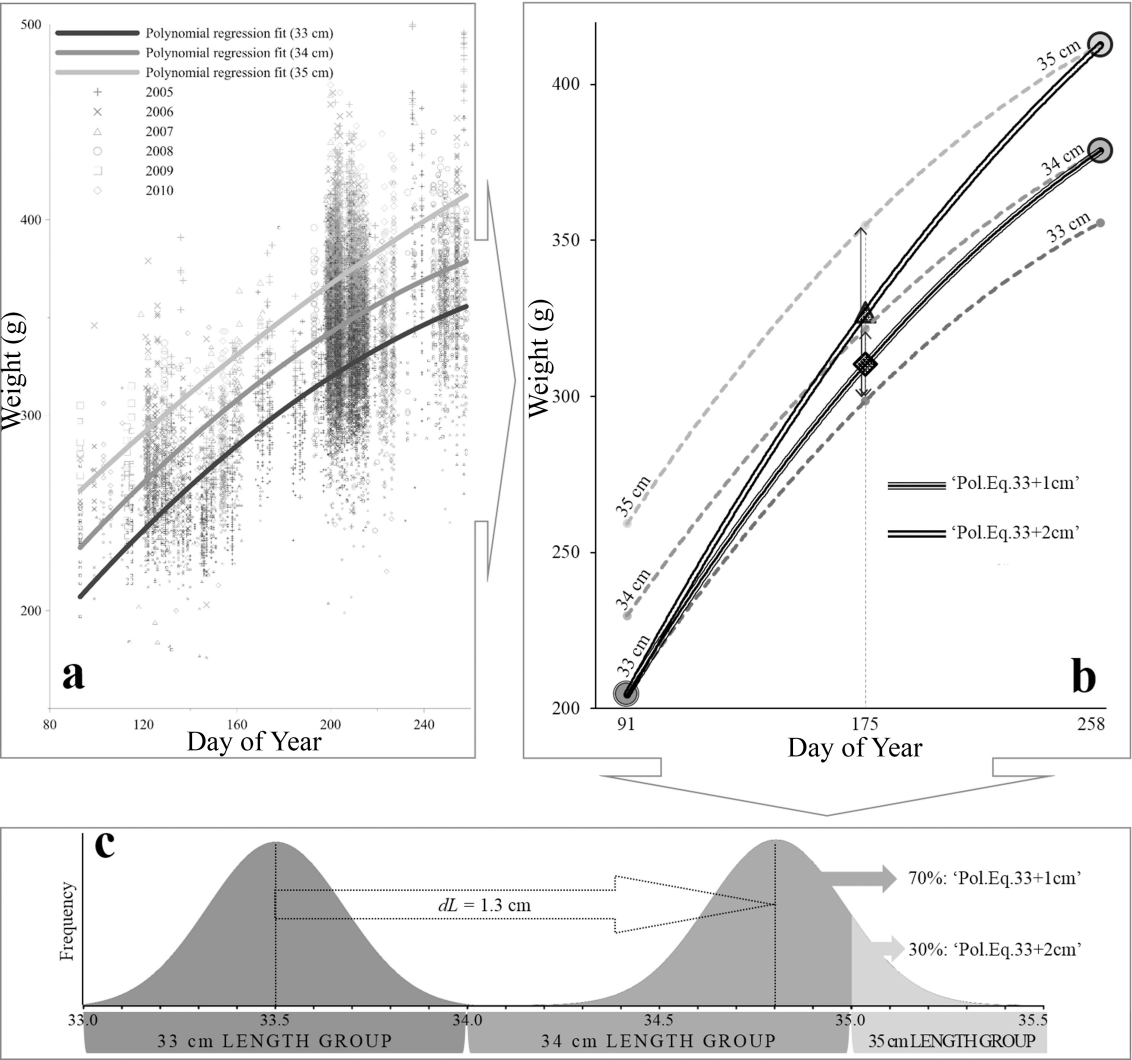
Example of growth correction for length distribution of herring, considering 33 cm length group as reference and a *dL* value of 1.3 cm (as a result example for Eq 6). Firstly, **(a)** polynomial equations are fitted for weight (g) increment per day (Day of Year) in 33, 34 and 35 cm length groups. Secondly, **(b)** considering the beginning of the feeding period (Day of Year = 91, marked as opened circle) as a starting point for the 33 cm length group, and the end of the feeding period (Day of Year = 258) for 34 and 35 cm length groups as ending points (closed circles), new equations are built for each cm growth steps: ‘Pol.Eq.33+1cm’ equation for 33+1 cm and ‘Pol.Eq.33+2cm’ equation for 33+2 cm. The mid-point (Day of Year = 175, marked with rhombus and triangle) in weight estimates for each new equation has the same distance (vertical arrows) from the estimated weight at that day for the lower length group and for the upper length group. Finally, **(c)** based on the *dL* and assuming a normal distribution for each cm length group, ‘Pol.Eq.33+1cm’ equation applies to the 70% of the 33 cm herring population abundance that will incorporate to the 34 cm length group one year later, whereas ‘Pol.Eq.33+2cm’ will apply to the other 30% that will correspond the 35 cm length group.

In order to ease the understanding of this adjustment, an illustrative example is presented in Fig 3, applying a *dL* of 1.3 cm for the 33 cm length group in herring. This means that part of the 33 cm herring population will grow to 34 cm, whereas the rest will reach 35 cm, in one year period. The regression parameters of daily increment in weight used as reference are those obtained from Eq (5) for fixed length groups (e.g. 33, 34 and 35 cm herring; Fig 3A). To correctly estimate the seasonal change in weight given the length growth, new polynomial equations for each cm length group and year were fitted, considering an increase in length defined by the lower (*dG*_*lower*_ = +1 cm) and upper (*dG*_*upper*_ = +2 cm) bounds for the estimated increment (*dL* = +1.3 cm). Each polynomial equation was obtained from three points: 1) the weight at a given length group at the beginning of the feeding season, 2) the weight at the end of the feeding season after applying a certain growth increment (amount of cm increments defined by *dG*_*lower*_ or *dG*_*upper*_ in each case), and 3) the mid-point between the two previous values (i.e. estimated weight in grams when Day of Year is 175) (Fig 3B). Each equation obtained was applied to the percentage of the population abundance within the corresponding length group after considering growth in one year period, (Eq 7), i.e. *RdL*_*lower*_ = 70% and *RdL*_*upper*_ = 30% (Fig 3C).

As stated before, the daily change in energy density (J g^-1^ fish) has to be also considered to determine the change in total energy for each individual fish during the feeding season.

**Fig 4:**
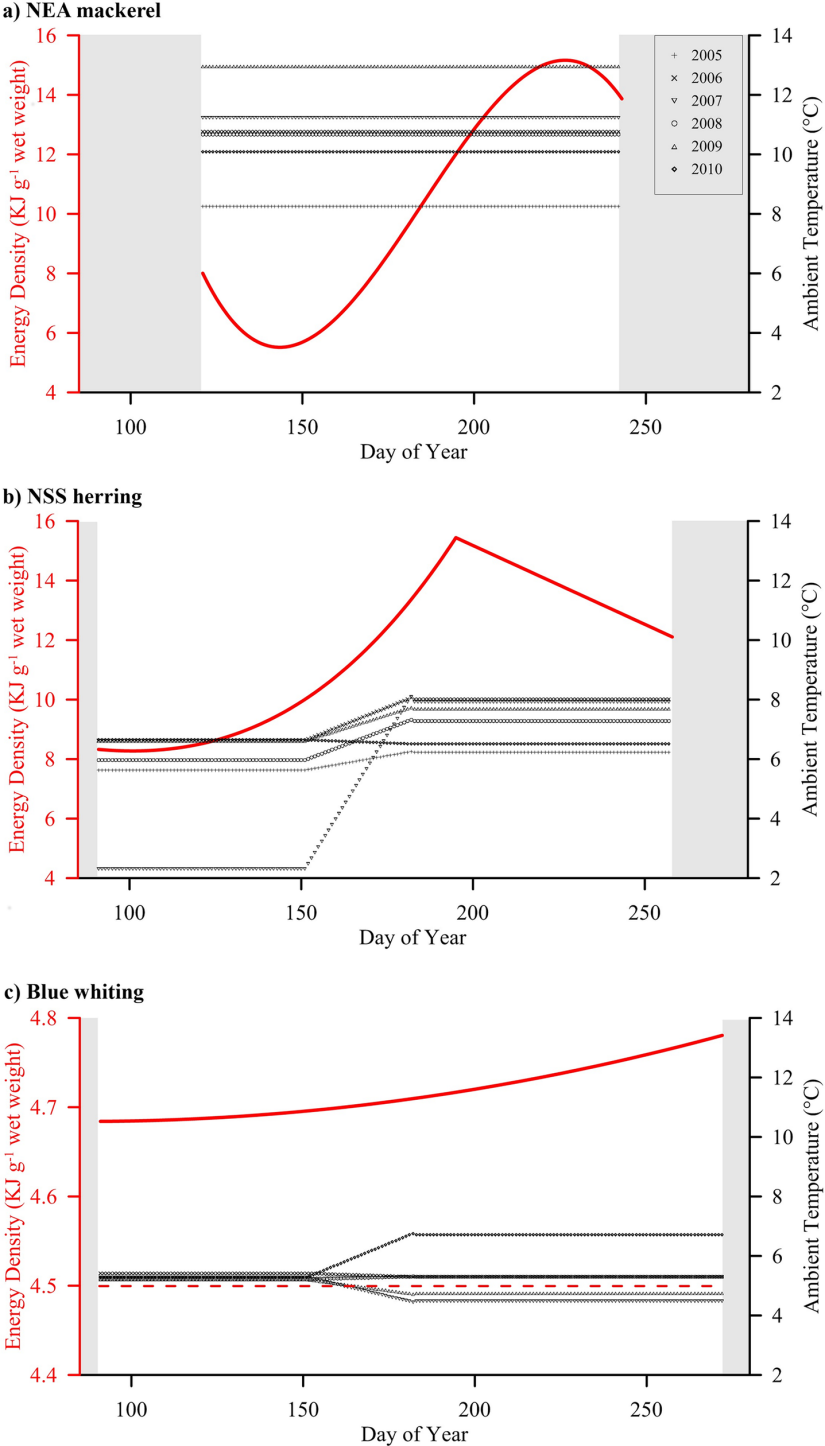
**Daily energy density averages (red lines and left Y axes, in kJ g**^**-1**^ **wet weight) and ambient temperature (black symbol-lines and right Y axes) for (a) NEA mackerel, (b) NSS herring (energy density as in Varpe et al.** [11]**) and (c) blue whiting.** White background represents the feeding period assumed for each species. The blue whiting plot (c) represents the assumed fixed value for cod (horizontal red dotted line) and the accumulated energy in liver (*E.Lv* = 0.0028*t*^2^ − 0.4842*t* + 205.02; *R*^*2*^ = 0.997); this equation was extracted from our model observations (note the different scale in left Y axis). See Table 5 for further details about equations used in the bioenergetics model.

The energy composition of mackerel changes substantially through the year. During spawning the average lipid content decrease to 2–8%, followed by a rapid increase in June–July to mid-summer, where it peaks at 25–31% [62–64]. The dry weight fraction of the wet weight follows the lipid content closely (R^2^ = 0.99), so other parts remain nearly constant. Seasonal changes in energy content of mackerel was expressed by a third degree polynomial model (Fig 4 & Table 5 –Eq(8)) fitted to the data (Dryad Digital Repository; doi: 10.5061/dryad.gb786).

**Table 5:** Equations and values used to estimate changes in energy density (*ED*) during the feeding period (*t*) for predator species (*pred*.*sp*): NEA mackerel, herring and blue whiting. For blue whiting, the energy content in liver (*E*.*Lv*) is added to the constant energy density of the fish muscles for gadoids (*ED*_*G*.*m*._), assumed in previous studies. *t*: Day of Year; *ED*_*t*,*pred*_: Energy Density of fish (*pred*: predator species) at day *t*. *E*.*Lv*: Energy content in liver (based on observations for Pollock, in kJ); *W*.*Lv*: Weight of the liver (g); *ED*.*Lv*: Energy Density in liver (J g^-1^); *L*_*ind*_: liver index; *W’*_*t*_: total weight of fish (as a function of time *t*); *DM*.*Lv*: Proportion of dry material in liver; *O*.*Lv*: Proportion of oil in liver.

Species (*pred*.*sp*)	Time period (*t*)	Equation (or value)	Reference
NEA mackerel	121–243 (May01 –Aug31)	*ED*_*t,pred*_ = −3.367 10^−5^*t*^3^ + 0.019*t*^2^ −3.282*t* + 191.171 (8)	this study
NSS herring	74–195 (March15 –July14)	*ED*_*t,pred*_ = 2.749 10^−6^*t*^3^ − 2.807 10^−4^*t*^2^ − 0.027*t* + 11.067 (9)	[11]
	196–259 (July15 –Sept15)	*ED*_*t,pred*_ = −0.053*t* + 25.771 (10)	[11]
Blue whiting	91–273 (April01 –Sept30)	*ED*_*t,pred*_ = *ED*_*G.m.*_ + *E.Lv* (11)	this study
		*ED*_*G*.*m*._ = 4500 J g^-1^	[65]
		*E.Lv* = (*W.Lv*)(*ED.Lv*) (11.1)	[68]
		*W*.*Lv* = *L*_*ind*_/100*W*′_*t*_ (11.1.1)	[67]
		$ED.Lv=\left[DM.Lv\left(39.55-\left(16.49e^{-0.235L_{ind}}\right)\right)\right]+39.55\left(O.Lv\right)$ (11.1.2)	[68]
		*L*_*ind*_ = 4 + 1/30.33*t* (11.2.1)	[68]
		*DM.Lv* = (1.085 − (0.824/*L*_*ind*_)) − 0.276*log*(*L*_*ind*_) (11.2.2)	[68]
		*O.Lv* = 0.276*log*(*L*_*ind*_) − 0.2 (11.2.3)	[68]

Daily estimates in energy density of NSS herring were based on data from Slotte [43], using the obtained regression equations (Eqs (9) and (10) in Table 5; Fig 4) as input for the bioenergetics model as in Varpe et al. [11].

For blue whiting there is no relevant information available, and we assumed the same energy density value as for Atlantic cod (*Gadhus morhua*) [65], given that these two species previously have been considered as comparable gadoid fish [13,66]. Gadoids do not accumulate energy in muscles, but store it in the liver throughout the feeding season [67,68]. On average the liver increases from 4% to 9% of the body mass in the period April to September [67]. Therefore, in order to incorporate that energy accumulation in the liver to the energy density values, in this study we applied the equations given by Dumke [67] (Fig 4 & Table 5 –Eq(11)).

### Consumption estimates from bioenergetics model

Table 6 summarizes the functions, variables and parameters need as input in the bioenergetics model (Eq 1). Results were presented in terms of energy consumption (Joules) and wet weight biomass (grams).

**Table 6:** Equations, variables and parameters used in the bioenergetics model, obtained from Elliott and Davison [71], Kitchell et al. [36], Hanson et al. [37], Hansson et al. [72], Stewart and Binkowski [45] and Stewart et al. [73]. All weights are wet weights (g).

Description	Equations, Variables and parameters	Values or reference equations (Eq.), Tables or sections (Sct), by predator species		
		NEA mackerel	NSS herring	Blue whiting
Consumption	*C* = *R* + *F* + *E* + *S* + *G*	Eq (1)	Eq (1)	Eq (1)
Respiration (metabolism)	$R=\alpha W^{\prime \beta }e^{\rho \;aT}e^{T_{opt}SW}$ (12)	Eq (12)	Eq (12)	-
	*R* = *αW*′^*β*^*V*^*x*^*e*^(*x*(1−*V*))^*act* (13)	-	-	Eq (13)
	*α*: Intercept of the allometric weight function (*RA*) corrected for the energy equivalent of Oxygen (J g^-1^ O_2_^-1^) and energy density of fish. $\alpha =RA\frac{13560}{ED_{pred}}$ (14)	Eq (14)	Eq (14)	Eq (14)
	*RA*: Intercept of the allometric weight function (g O_2_ g^-1^ day^-1^)	0.00264	0.0033	0.008
	*ED*_*pred*_: Energy density of (predator) fish	Table 5	Table 5	Table 5
	*W’*: Fish weight (body mass, g)	Eq (7)	Eq (7)	Eq (7)
	^*β*^: Slope of the allometric weight (*W'*) function	-0.217	-0.227	-0.172
	$V=\frac{T_{max}-aT}{T_{max}-T_{opt}}$ (15)	-	-	Eq (15)
	$x=\frac{{\left[\ln\rho \left(T_{max}-T_{opt}\right)\right]}^{2}{\left[1+{\left(1+40/\ln\rho \left(T_{max}-T_{opt}+2\right)\right)}^{0.5}\right]}^{2}}{400}$ (16)	-	-	Eq (16)
	*ρ*: Slope for temperature (*aT*) dependence (-°C^-1^); approximates the rate at which the function increases over relatively low water temperatures	0.06818	0.0548	1.88
	*aT*: Ambient temperature (°C)	Eq (2)	Eq (2)	Eq (2)
	*T*_*max*_: Maximum (lethal) water temperature (°C)	-	-	24
	*T*_*opt*_: Optimal temperature (°C) as slope for swimming speed (SW) dependence	0.0234	0.03	21
	*act*: The Winberg activity multiplier	-	-	1.25
	*SW*: Swimming speed (body length s^-1^)	1	1	1
Egestion	*F* = *θ* *C* (17)			
	*θ*: Proportion of consumed food egested	0.16	0.16	0.17
Excretion	*E* = *ε* (*C* − *F*) (18)			
	*ε*: Proportion of assimilated food excreted	0.10	0.10	0.09
Specific dynamic action	*S* = *ω* (*C* − *F*) (19)			
	*ω*: Coefficient, specific dynamic action	0.172	0.175	0.17
Growth	Body mass change per length group (as a function of time *t*): $G\left(t\right)=\frac{\left(W{´}_{\left(t\right)}ED_{pred\left(t\right)}\right)-\left(W{´}_{\left(t-1\right)}ED_{pred\left(t-1\right)}\right)}{ED_{pred\left(t\right)}}$ (20) $G\left(t\right)=\frac{\left[\left(W{´}_{\left(t\right)}ED_{G.m.\left(t\right)}\right)-\left(W{´}_{\left(t-1\right)}ED_{G.m.\left(t-1\right)}\right)\right]+E.Lv}{ED_{pred\left(t\right)}}$ (21)	Eq (20) -	Eq (20) -	- Eqs (11,21)

The software packages *R v*. *3*.*0*.*2* [69] and *ggplot2 v*. *1*.*0*.*0* [70] were used for data analysis and graphical representations, respectively. Fig 1 was plotted with package *mapdata v*.*2*.*2–6* and Fig 3 was made with *Grapher v*. *8*.*2* software. The bioenergetics model was run in *Fortran*, using *Eclipse v*. *Neon Milestone 2* for Parallel Application Developers (www.eclipse.org).

## Results

### Prey consumption estimates

**Fig 5:**
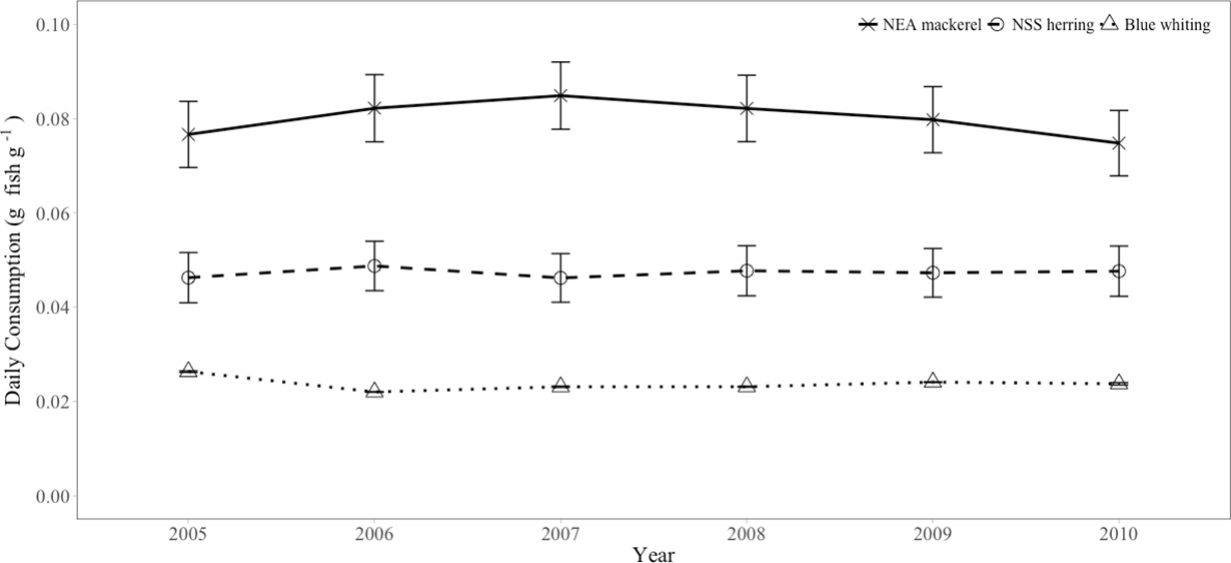
Average daily consumption estimates ±2SE (prey g fish g^-1^ day^-1^) by NEA mackerel, NSS herring and blue whiting, per year (from 2005 to 2010).

The specific average daily consumption rates (Fig 5) did not show any significant inter-annual variability (Tukey HSD test, p > 0.05). Average values could therefore been considered over the entire time series. The species with the shortest feeding season, NEA mackerel, had the highest mean daily consumption rate of 0.08 g prey g fish^-1^ day^-1^. NSS herring consumed 0.05 g prey g fish^-1^ day^-1^, whereas blue whiting showed the lowest value, 0.02 g prey g fish^-1^ day^-1^ (Tukey HSD test p < 0.001 for the three paired comparisons). This is equivalent to a daily consumption rate of 8% of fish body weight for NEA mackerel, 6% for NSS herring and 2% for blue whiting.

**Fig 6:**
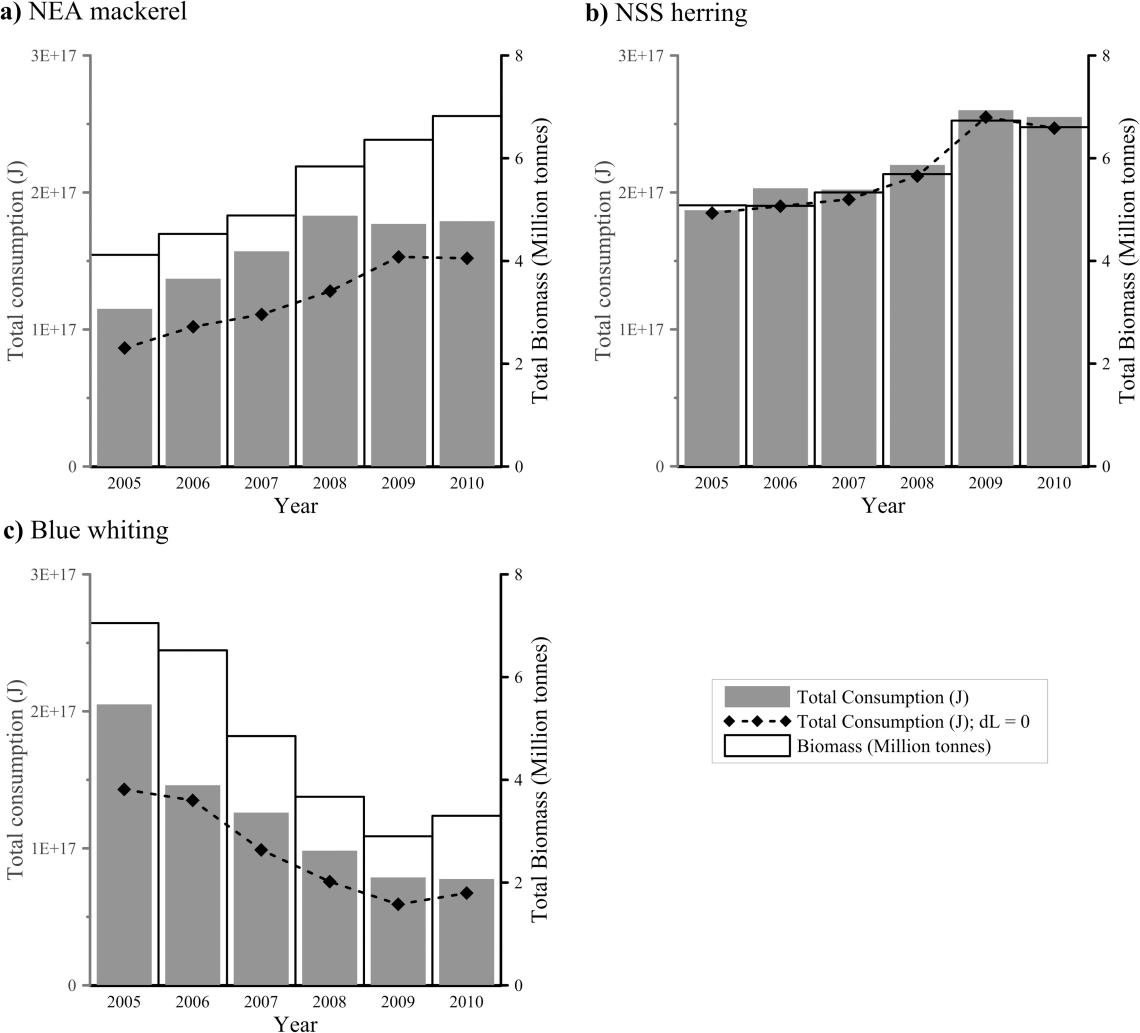
Total annual (2005–2010) energy consumption (Joules) by NEA mackerel, NSS herring and blue whiting, indicated as dark grey bars (left vertical axis). Empty bars (based on right vertical axis) indicate the total biomass (in Million tonnes) from the assessment (TSB for NEA mackerel and blue whiting, SSB for NSS herring; [9]). Dotted line represents consumption estimates when the daily (somatic) growth of fish was set to 0 in the bioenergetics model, as assumed in Utne et al. [13] and Varpe et al. [11].

The total annual prey consumption by the NEA mackerel and NSS herring stocks generally increased during the study period from 2005 to 2010 (Fig 6) while it decreased for blue whiting stock (Tukey HSD test p < 0.001 for both interspecific and inter-annual variability). Inter-annual variation in total consumption primarily reflected variation in predator biomass (Fig 6). However, in 2009–2010, when the total biomass of NSS herring and NEA mackerel were the highest, the total energy consumption of NEA mackerel remained stable or even decreased despite an increasing stock size. NSS herring consistently showed higher total consumption than NEA mackerel, even in 2010, when the biomass of NEA mackerel exceeded NSS herring (Fig 6).

**Fig 7:**
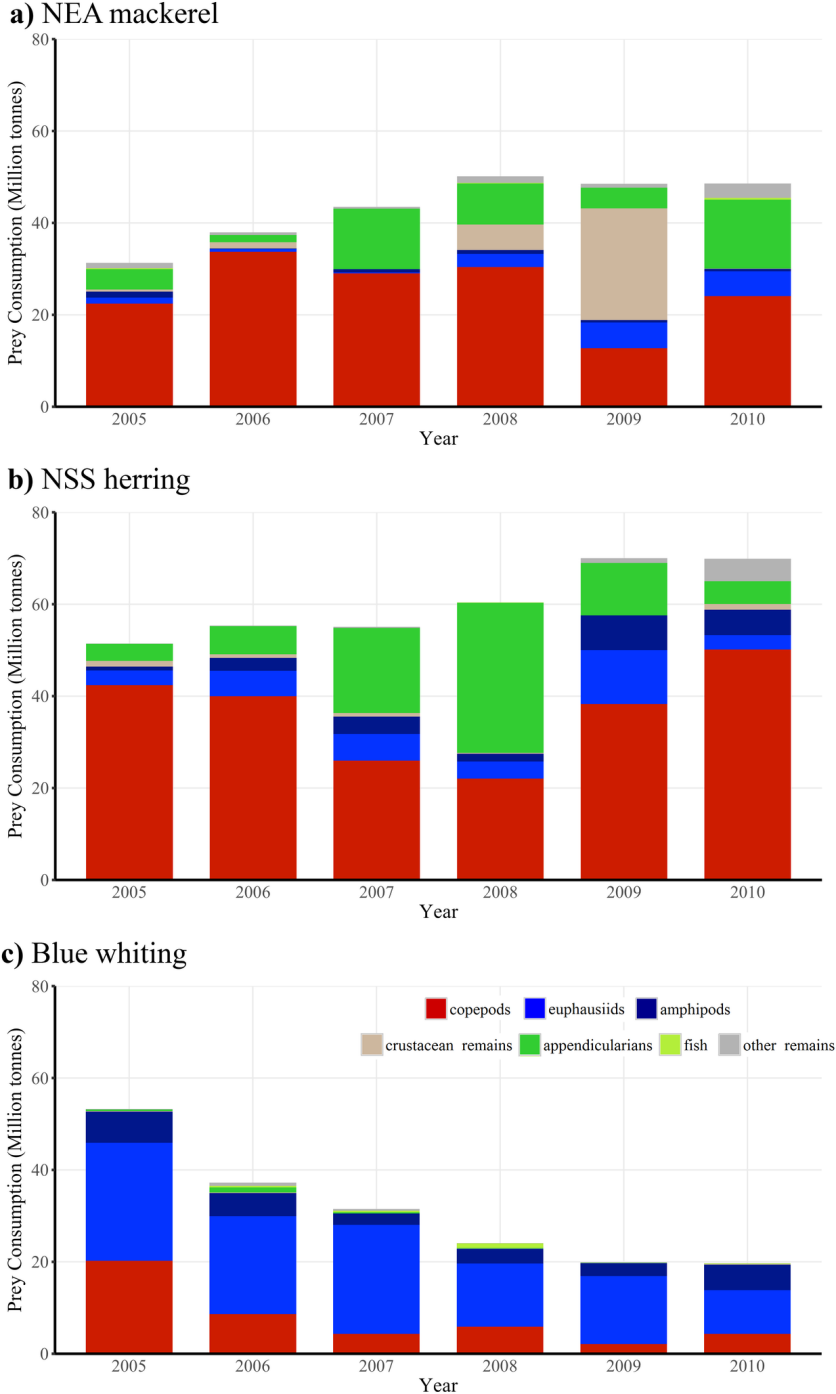
Annual (2005–2010) prey consumption estimates for NEA mackerel, NSS herring and blue whiting.

Regarding the annual zooplankton consumption, our bioenergetics modeling resulted in estimates ranging from 31 to 51 million (M) tonnes by NEA mackerel, 51–70 M tonnes by NSS herring and 20–53 M tonnes by blue whiting (Fig 7). Depending on the year, NEA mackerel consumed 23–38% of the total zooplankton eaten by the three pelagic fish species during the feeding season (24–52% of the copepods and 2–30% of the euphausiids eaten); NSS herring consumed 38–51% (38–72% of the copepods and 11–36% of the euphausiids eaten), and the blue whiting consumed 14–39% (4–24% of the copepods and 46–85% of the euphausiids eaten).

**Fig 8:**
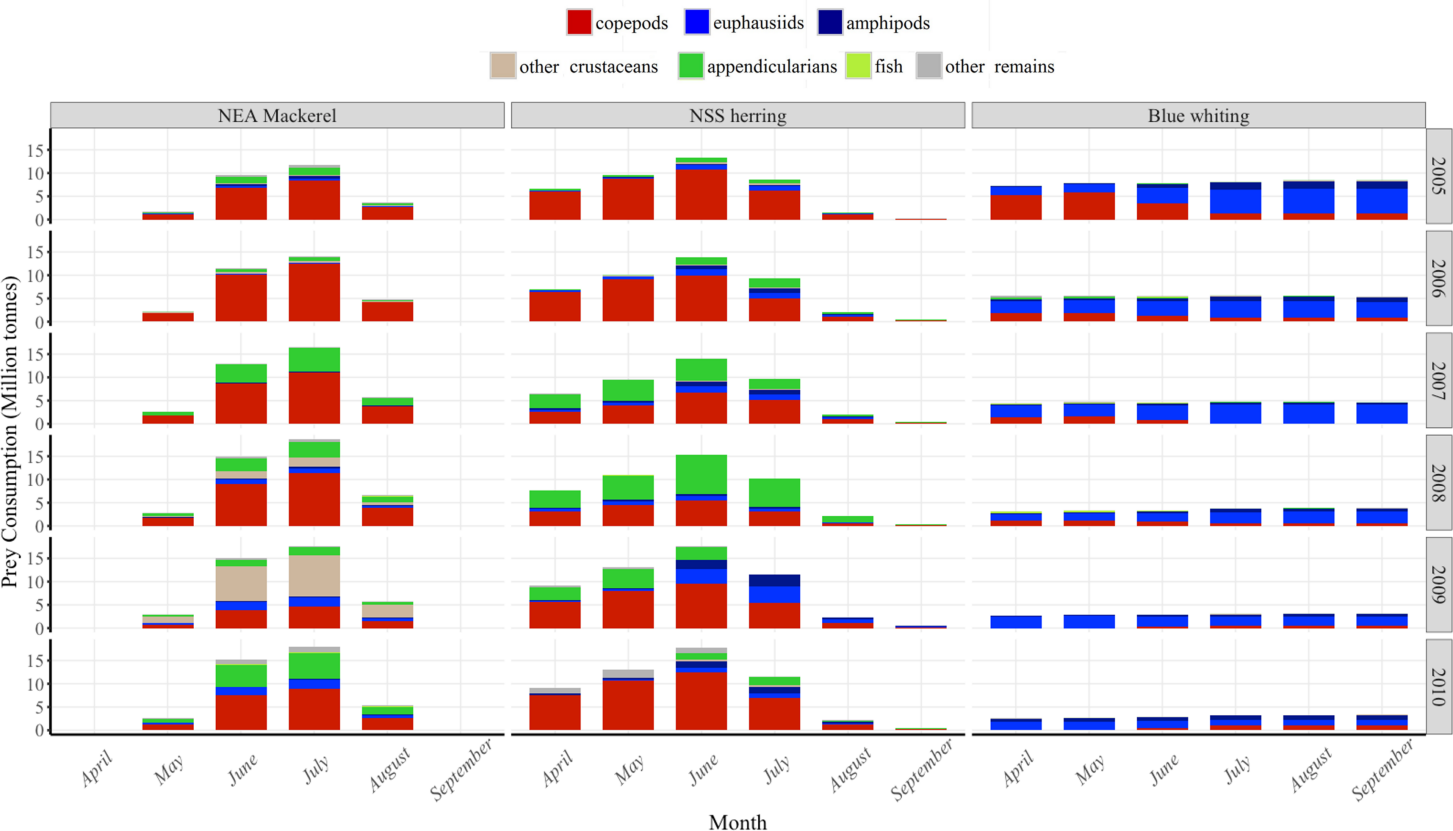
Seasonal variation in prey consumption by NEA mackerel, NSS herring and blue whiting during their species-specific feeding periods in 2005–2010.

According to the obtained estimates from our model, NSS herring showed high levels of zooplankton consumption in both spring and summer, gradually increasing from April to June, followed by a slight reduction in July. Very little feeding was done in August and September (Fig 8). The modeling results suggest that mackerel fed intensively in the Norwegian Sea in June and July, followed by lower consumption rates in August. In contrast, blue whiting maintained an almost constant low consumption rate through entire feeding season (Fig 8).

### Total consumption of major prey groups

The most important prey for NEA mackerel and NSS herring were copepods (especially calanoids [8,57]), and euphausiids for blue whiting (Fig 7). However, substantial inter–annual variation was indicated. For instance, appendicularians and other crustaceans (mainly cladoceran *Evadne* spp. [8,57]) were also important, or even dominating, in the diet composition of NEA mackerel and NSS herring in some years. Copepod consumption by NSS herring decreased from 2006 to 2008, copepods being partially replaced by appendicularians, which consisted in more than 50% of the diet composition in 2008 (Fig 7). This pattern was reversed in 2009 and 2010. Copepod consumption by blue whiting also decreased from 2005 onwards, being replaced by larger prey like krill or early life stages [8] of fish (e.g. 2006–2008, Fig 7).

Considering the three species together, they were estimated to have consumed 53–81 M tonnes of copepods, 26–39 M tonnes of euphausiids and amphipods, 8–42 M tonnes appendicularians and 0.2–1 M tonnes of fish, depending on the year (Fig 7).

The diet composition of NEA mackerel was only sampled during summer and seasonal changes could therefore not be analyzed. For NSS herring and blue whiting, some changes were apparent. For example, NSS herring fed mainly on copepods and appendicularians early in spring while euphausiids and amphipods entered the diet during summer (Fig 8). Blue whiting fed mainly on copepods in high percentages of their diet early in spring and early summer, shifting to a more selective diet based on euphausiids and amphipods later in the season (e.g. in 2005, Fig 8).

### Consumption–Predator biomass ratios

**Fig 9:**
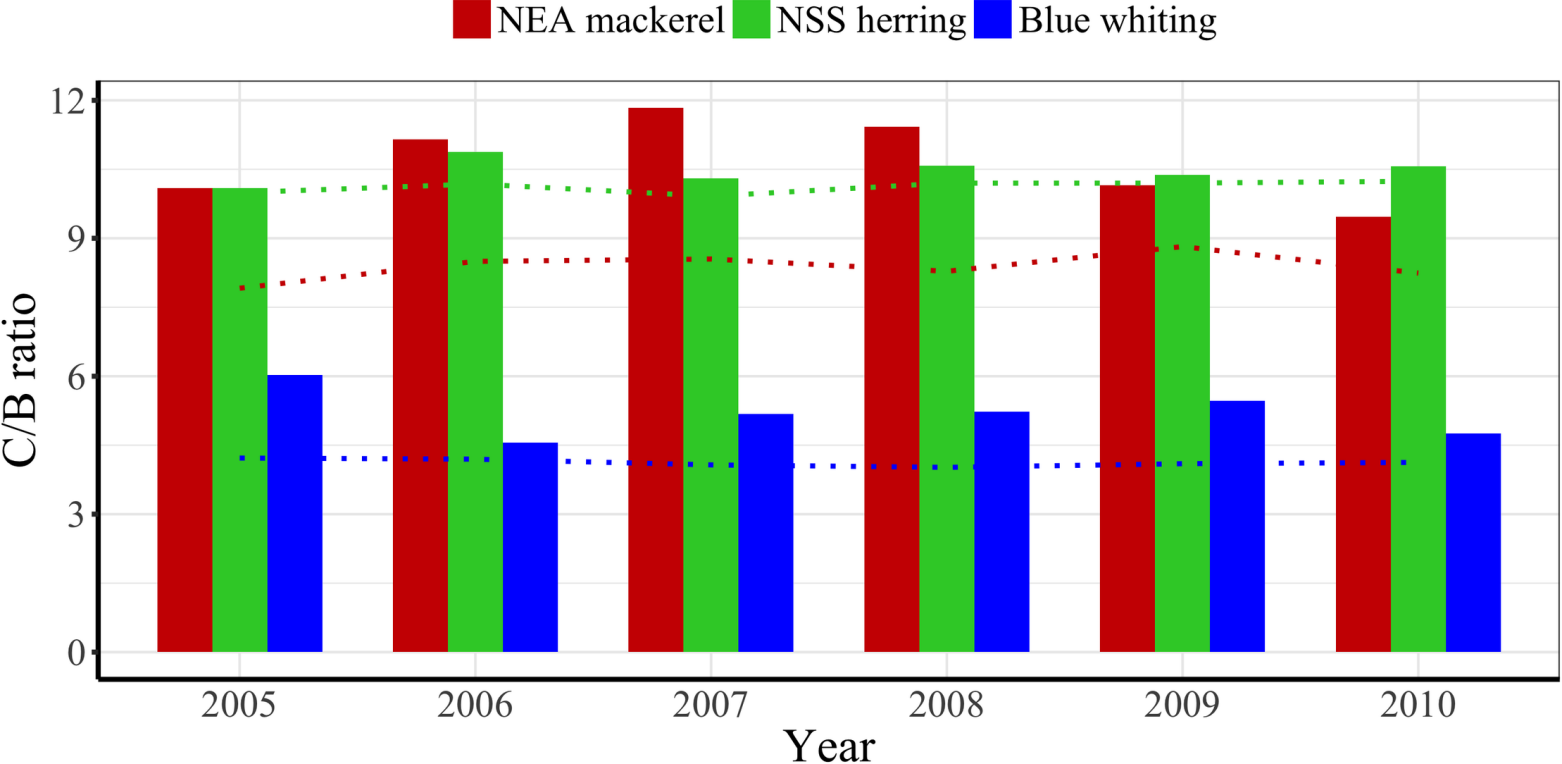
Consumption/Biomass ratios per year and species. Dotted lines represent estimates when daily growth of fish is not considered [11] in the model.

Total annual consumption (in prey weight) relatively to the biomass of the stock (C/B ratio) differed between the predators. NEA mackerel and NSS herring were at a similar level consuming between 9.5 and 12 times their biomass in zooplankton (Fig 9). Blue whiting was estimated to consume around the half, with a C/B ratio between 4.7 and 6 (Fig 9).

**Fig 10:**
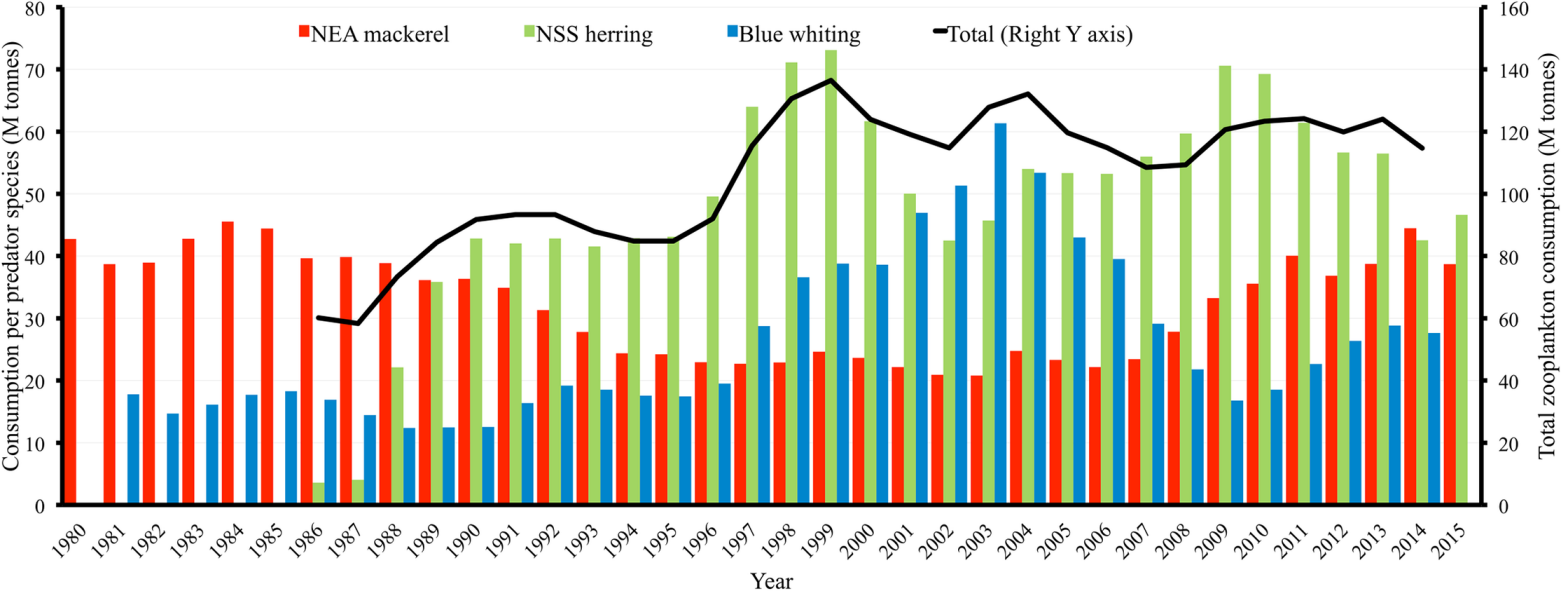
Total zooplankton consumption by NEA mackerel, NSS herring and blue whiting, from 1980 to 2015. Estimates are based on the mean consumption values from 2005–2010, which were then extrapolated to the total fish biomass reported for different years by the assessment [9]. Black line represents the total consumption by the three species (note the different scale on the right vertical axis).

The low inter-annual variation in C/B ratio could indicate that the mean C/B ratio for the period 2005–2010 could be used to extrapolate into unobserved years. Based on this assumption, and ICES assessments of total fish biomass [9], the total zooplankton consumption was estimated from 1960 to 2015. This suggested that the three predators had approximately consumed between 100 and 130 M tonnes of zooplankton annually in the last two decades (Fig 10). In addition, our estimates suggest that NSS herring had consumed most zooplankton in almost all years since 1990. Blue whiting consumed more zooplankton than NEA mackerel during the years where this stock was highly abundant (1998 to 2006). Zooplankton consumption by NEA mackerel has increased from 2007 and levelled out from 2011 to 2015 (Fig 10).

### Parameter sensitivity on consumption estimates

**Fig 11:**
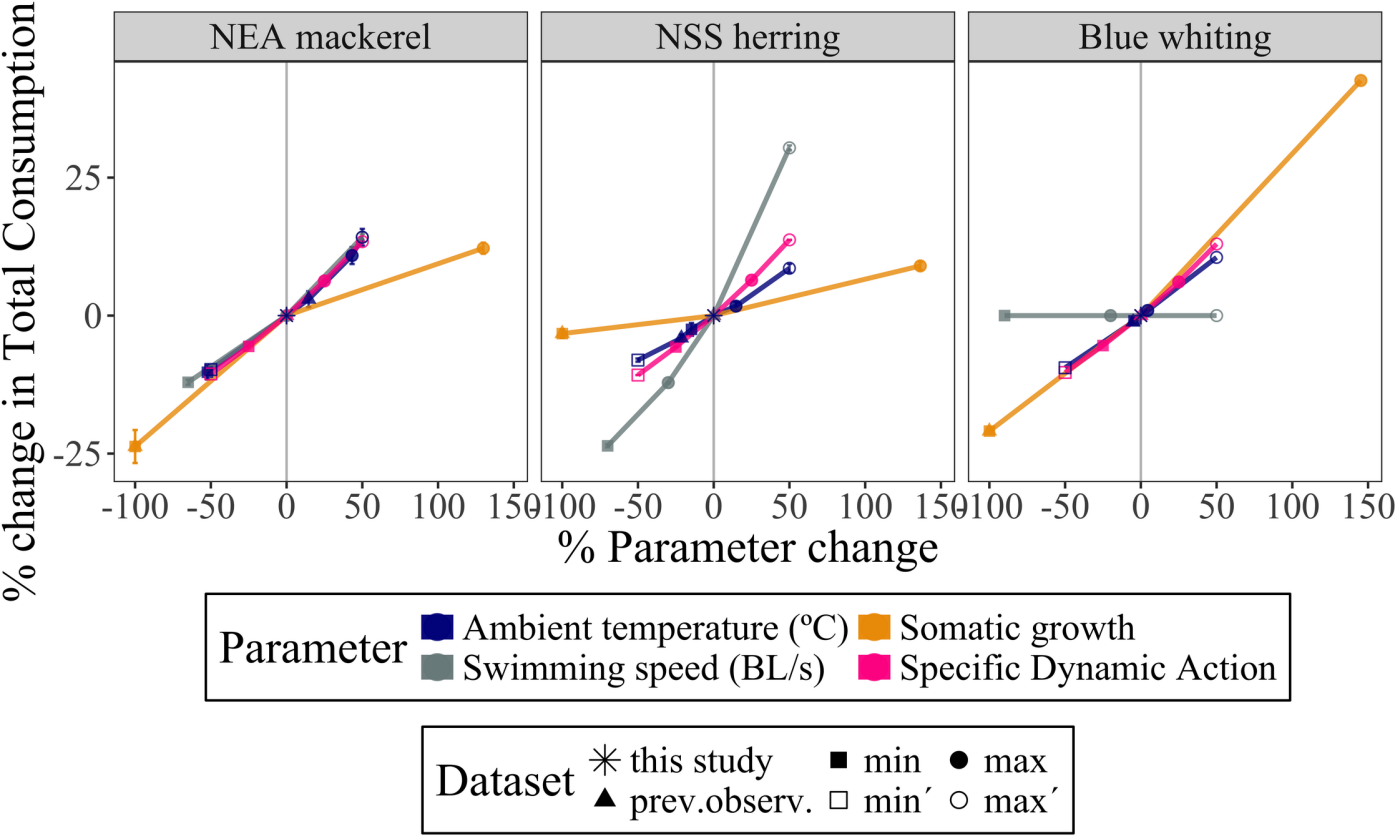
Sensitivity analysis of the bioenergetics model, presented by spider diagrams showing the percentage of variation in estimates of the total energy consumption (J) by NEA mackerel, NSS herring and blue whiting, depending on the percentage of change of different parameter variables: ambient temperature (°C), swimming speed (body length s^-1^), somatic growth and coefficient of the specific dynamic action (*ω*). The mean %change value for the five years of the analysis (2005–2010) is represented with error bars indicating the standard error. Symbols indicate different assumptions made when determining parameter values, based on different sources. *this study*: values as used in the present study (i.e. % change of parameters = 0); *prev*.*observ*: values used as in previous studies (e.g. Utne et al. [13] and Varpe et al. [11]); *min* and *max*: minimum and maximum possible values mentioned in previous literature, respectively; *min’* and *max’*: artificial (unrealistic) values used to highlight the general changing trend of estimates when extreme values are considered.

Fig 11 represents a sensitivity analysis to show how the final consumption estimates were affected by different parameter values assumed in the bioenergetics model. For the three species the ambient temperature, swimming speed, daily (somatic) growth and specific dynamic action showed a positive relation with the total consumption estimates, i.e. higher parameter values resulting in higher estimates. Increasing the swimming speed leads to higher metabolic rate, which increases the energy loss due to respiration. The effect of changing the growth and swimming speed values in the model for NSS herring and blue whiting consumption estimates, respectively, was lower, in comparison with the other species (Fig 11).

## Discussion

In contrast to previous studies estimating zooplankton consumption by pelagic planktivores in the NEA Atlantic, the current study incorporates more detailed information on diet composition, ambient temperature and length–growth during the feeding migration. Accordingly, the bioenergetics model presented in this study estimated that the three stocks on average consumed 135 mill tones of zooplankton in the study period. Our consumption estimates are higher than in previous literature for the same stocks. In all sampling years NEA mackerel showed higher daily consumption rates than the other two stocks, whereas the amount of prey consumed by blue whiting was relatively lower. Given that the variation in annual consumption seems to be mainly driven by the total fish biomass, our results showed that 14.70–17 M tonnes of pelagic fish consumed between 131 and 139 M tonnes of zooplankton in 2005–2010, which is more than the consumption estimated by Utne et al. [13] and moreover the double of that estimated by Skjoldal et al. [1] for similar amount of fish. Considering the three species together, Skjoldal et al. [1] and Utne et al. [13] determined a C/B ratio of 3.75 and 6.3 respectively, while our calculations yielded a C/B ratio ranging from 7.87 to 9.21, depending on the year. This is a rather large difference. In fact, when assuming no length–growth and applying a constant temperature of 5°C as in Varpe et al. [11], consumption estimates obtained by our model for NSS herring are close to their estimates, ranging from 1.47 (10^17^) J to 1.96 (10^17^) J (e.g. see dl = 0 in Fig 6). However, even in such case, obtained C/B ratios (7.88–7.96) are more than 50% higher in comparison with values assumed in Dommasnes et al. [12]. The most important difference regards in the inclusion of combined individual length growth (i.e. somatic growth), changes in weight-at-length and changes in energy density of the fish during the feeding season. By combining all these factors a realistic estimate of the amount of energy stored as muscles or fat in the fish can be achieved, and thus how much the fish must have consumed to achieve the observed growth. Earlier work has either ignored the change in energy density (e.g. Utne et al [13]), the increase in length growth (e.g. Varpe et al. [11]), or used a simpler general approach to estimate the total consumption of pelagic fish in the Norwegian Sea (e.g. Skjoldal et al. [1]).

Bioenergetics modeling has several uncertainties related to the parameter estimates and the functional relations [38]. The model relies on quite a few parameters, some of which are based on experimental work on different species than applied here. This may either lead to bias in consumption estimates as there are differences between species in energy cost associated with movement, digestion of prey, and how this vary with water temperature. Accordingly, it should be noted that the ambient temperature was calculated taking into account the spatial distribution of the fish, resulting in more accurate but slightly higher temperatures than assumed in previous studies, which therefore resulted in higher zooplankton consumption estimates (Fig 11) due to higher respiration costs [47,48]. Besides, assumed swimming speed had an important effect in consumption estimates for NSS herring and (especially) NEA mackerel (Fig 11). The parameter used for the specific dynamic action (Table 6, Fig 11) as well as other factors such as natural mortality [74] or seasonal [46] and inter-annual variations in diet composition [8,74] also influenced consumption estimates, although larger time-series would be needed to reduce the uncertainty of using bioenergetics modeling (e.g. [75]) as well as to make further conclusions.

The generally higher contribution of copepods and lower contribution of euphausiids in NEA mackerel stomachs than in NSS herring stomachs, as well as the different diet of the blue whiting were well reflected in our consumption estimates, in accordance with previous stomach content investigations [6,8,76,77]. In fact, the diet of small pelagic fish changes with prey availability [6–8,78,79]. This way, the three fish species are abundant with overlapping diet and are able to coexist in the same habitat, due to a certain degree of niche differentiation [8]. NEA mackerel and blue whiting have little vertical overlap [52,80] and different ability to filtering small zooplankton [6,8]. This is reflected in the annual consumption estimates, as blue whiting mainly prey on euphausiids while this prey is of minor importance for mackerel. So while NEA mackerel feed on small zooplankton like copepods and appendicularians close to the surface, blue whiting selectively pick larger zooplankton at 100–400 m depth. On the other hand, NSS herring and NEA mackerel have large vertical overlap as they both feed close to the surface in the feeding period [8,52,80]. For these two species the niche differentiation is also evident as NSS herring consume copepods and appendicularians early in the feeding season before mackerel enter the feeding grounds, but switch to euphausiids and amphipods later in the summer [8]. This separation may partly be explained by horizontal segregation as NSS herring migrate into colder waters (2–6°C) further north and west than NEA mackerel [4,51], but other mechanisms such as the potential inter-specific feeding competition [8] are probably also important, as there are large areas where the two species horizontally overlap. Differences in the degree of particulate feeding (i.e. selective predation) and filter feeding, or in the amount of daylight needed for efficient feeding may influence the total consumption estimates. The large consumption of euphausiids and amphipods by NSS herring late in the summer suggest that (1) they might base their feeding strategy on selective predation, which is reasonable in areas with limited prey available [81], or (2) they could feed in the dark [82], when the large zooplankton raise towards the surface [1].

The target stocks are spread over large areas in the North Atlantic and it is difficult to attribute the predation to particular areas such as the Norwegian Sea. For instance, NEA mackerel might have been incompletely covered by our model and data collection during spring, when it was spawning and probably also feeding south of our study area [9]. Nonetheless, considering all the NEA mackerel older than 2 years feeding within the Norwegian Sea and according to the diet description based on adults caught in that area [8] might also cause some kind of bias. Besides, the proportion of the NEA mackerel stock feeding in the North Sea and further south is not known. By combining estimated biomass from surveys in the North Sea [83] and the Norwegian Sea [42], and assuming only minor abundance not covered by these two surveys, we can estimate that 10–30% of the total NEA mackerel stock was feeding in the North Sea in 2007–2010. However, it must be pointed out that there is a high uncertainty around this proportion, as for instance the surveys may have a very different catchability, as well as regarding the mackerel feeding in further southern areas (e.g. Bay of Biscay [9,78]). Nevertheless, even in the case we assume a more conservative proportion of the NEA mackerel stock feeding in the Nordic regions or at least passing through the area at any rate of their life cycle, the zooplankton consumption by this species seems higher than suggested in previous studies, especially for NEA mackerel. In fact, for NSS herring, the estimated relative zooplankton consumption (38–51% of the total zooplankton eaten by pelagic species) was lower than the 61% estimated by Utne et al. [13] for 1997. This is likely a result of the increase in NEA mackerel feeding in the area in recent years (Fig 10; [42]). Accordingly, Óskarsson et al. [77] also estimated that NEA mackerel consumed around 2.4–4.5 M tonnes in Icelandic waters during summer 2011, which seems low (given the proportion of mackerel in Icelandic waters [84]) compared to our consumption estimates for this species in the whole distribution area (31–51 M tonnes). The relative high total zooplankton consumption observed for NEA mackerel might also explain the strong density dependence seen in their growth [29] in recent years associated with the expanding stock size and distribution area [30]. This way, density dependence is incorporated in the model by reduced individual length and weight growth, according to observed annual growth for the time period handled in the model.

On the other hand, earlier consumption estimates of zooplankton made for the Norwegian Sea assumed that stomach samples taken during May–July are representative for the whole feeding season [1,11,12]. However, Utne et al. [13] suggested that planktivorous fish have relatively important changes in their diet composition during their feeding seasons as, for example in case of NEA mackerel and blue whiting, *C*. *finmarchicus* copepods are only available for them during parts of their feeding season. In order to approach the diet variation during the feeding season, the present study considered the diet composition in May and July as reference [8] and makes a linear interpolation to estimate the diet for the dates in between. This information is still limited, e.g. by the lack of NEA mackerel information from May or the spatial coverage of the stomach sampling, as commented before regarding the proportion of the total stock feeding within the Norwegian Sea. Moreover, our estimates can also be biased due to the diet information, as in general softer organisms (e.g. appendicularians) are digested more rapidly. In general softer organisms are digested more rapidly, which can lead to underestimation of these prey in the diet [8]. Bias in the diet can lead to wrong consumption estimates of certain prey species. In any case, it seems clear that the three species are exerting a significantly higher predation impact on the *C*. *finmarchicus* population than suggested in earlier studies (e.g. [11,13]) and therefore potential ecological implications (e.g. top-down control [1,75]) should be taken into account in the near future. Due to the potential and observed interactions between the three stocks [3,56,85] it has been proposed that the stocks should be managed as a multispecies system. A report being developed jointly by the Ministry of Fisheries, the Fisheries Directorate and the Institute of Marine Research suggests that the three stocks are candidates for multispecies management, but that more research and model development is needed before attempting to establish a management system.

Regarding the timing of the main consumption, it should be noted that during May only NSS herring and blue whiting are feeding in the south-central Norwegian Sea [13]. In early summer, these species generally move towards Northwest, while the NEA mackerel enters from the South [13]. This means that in July/August a large part of the Norwegian Sea is inhabited by pelagic fish, although the main predation pressure on zooplankton could be expected in the northern area, where NSS herring dominates. Also in this period, the total consumption by NEA mackerel appears to be higher than that by NSS herring. Utne et al. [13] defined the peak of consumption by NSS herring to be in April, whereas our results suggest the peak of feeding for NSS herring a bit later in the season, in June. This peak of feeding is also in accordance with observations based on light and feeding ecology made by Varpe and Fiksen [86]. For NEA mackerel, our results showed the peak of consumption in July, a month earlier than that suggested by Utne et al. [13]. This is relevant since it could increase the potential interactions between these species. In this sense, it is unclear whether the NSS herring makes a shift in the prey search from copepods towards euphausiids due to feeding interactions with NEA mackerel in summer [8]. Observed inter-annual differences in prey consumption can suggest a potential competition for prey between NEA mackerel and NSS herring mainly during July and early August, given that in years when one species consumes more copepods and less of other prey (e.g. appendicularians), the other species shows the opposite trend (Figs 8 and 9). However, such potential trophic interactions between these species should be further investigated due to the uncertainty regarding the spatial overlap during the feeding season [4,8,51,77].

Finally, the low seasonal and inter–annual variation observed in blue whiting agree with previous studies (e.g. Utne et al. [13]). This also suggests low interaction with the other species, probably due to a different vertical distribution and/or diet preference [8,51], more selective on euphausiids and amphipods, which at the same time would result in generally lower consumption rates. In addition, sampling biases regarding this species [8] could also affect the quality of input data to some extent.

## Concluding remarks

Our results suggest much higher annual zooplankton consumption by NSS herring, NEA mackerel and blue whiting than described previously. The higher consumption estimates are both due to higher biomass of pelagic fish than that assumed in previous studies, and due to higher consumption estimates obtained for the individual fish. In the time period 2005–2010, NSS herring and NEA mackerel consumed annually around 10 times their total biomass in zooplankton, mainly copepods, krill and appendicularians. Blue whiting consumed 6 times their biomass in zooplankton, with a diet mainly based on euphausiids. Between the three species it is estimated that their annual zooplankton consumption could be around 135 million tonnes.

To what extent the top-down and/or bottom-up control mechanisms are playing a role as regulation mechanisms of the pelagic ecosystem in the Northeast Atlantic is still unclear. In this sense, previous studies showed that the growth of blue whiting and in particular of NSS herring appeared to be negatively affected, especially by the increase of NEA mackerel biomass, through interspecific competition [3]. According to that, although the high consumption rates observed for both NSS herring and NEA mackerel during the feeding period as well as the shift in the diet of NSS herring later in the season when NEA mackerel is incorporated into the area might suggest some kind of interspecific feeding competition, it seems that the three species can coexist regardless of their high abundance, consumption rates and overlapping diet. Accordingly, our results show that the species might have niche segregation, as they are species specific, showing annual and inter-annual variability in total consumption of the different prey species. However, the potential consequences that the high amount of prey consumed by the three species could have as foragers in the zooplankton community, especially the *C*. *finmarchicus* population, remain unknown and should be taken into consideration for further research. This highlights the importance to understand marine trophodynamics in the context of the potential impact of pelagic fish consumption for the zooplankton community and its relevance for the pelagic fish populations through interspecific trophic interactions.

Annual estimates of diet require stomach sampling with good spatial and temporal resolution. Obtaining such good data is expensive and time-consuming. The results presented here should be sufficient for parameterization and calibration and of large-scale ecosystem models. Models such as Ecopath with Ecosim, Atlantis or coupled individual based ecosystem models can both hind-cast and forecast annual consumption estimates, and future effort should be put into improving such models.
